# Cure of Goiter by Fluoric Acid

**Published:** 1881-06

**Authors:** 


### CURE OF GOITER BY FLUORIC ACID.

Dr. Edward Woakes gives, in the *Lancet*, a detailed account of a number of cases of goiter cured by fluoric acid internally. He begins treatment with fifteen minims of a one-half per cent. dilution of the acid three times a day, and. if necessary, increases the dose to twenty, thirty, forty, or even seventy minims, and extends the time to several months. His results are quite remarkable, even in cases that had resisted iodine, bromine, iron, etc. In a few it was conjoined with injections of tinct. iodine. Very few failed to be reasonably benefited, and in eighty-five per cent. the cure was decided.

